# Lokale Strahlentherapie der Prostata mit Samenblasenbasis allein oder mit elektiver Bestrahlung pelviner Lymphknotenstationen beim Hochrisiko-Prostatakarzinom? Ergebnisse der randomisierten POP-RT-Studie

**DOI:** 10.1007/s00066-021-01825-x

**Published:** 2021-09-02

**Authors:** Martin Stuschke, Boris Hadaschik

**Affiliations:** 1 Klinik für Strahlentherapie, Universitätsklinikum Essen, Hufelandstr. 55, 45147 Essen, Deutschland; 2 Klinik für Urologie, Universitätsklinikum Essen, Hufelandstr. 55, 45147 Essen, Deutschland

## Hintergrund

Die elektive Strahlentherapie der Lymphabflusswege wird zusammen mit der Langzeit-Hormonentzugstherapie (ADT) seit mehr als 25 Jahren beim lokal fortgeschrittenen oder lokalisierten Prostatakarzinom der Hochrisikogruppe in randomisierten Studien untersucht [1, 2], aber bis heute kontrovers beurteilt [3].

## Patientenkollektiv und Methodik

Eingeschlossen wurden in die POP-RT-Studie von Murthy et al. (2021) im Rekrutierungszeitraum von 2011–2017 Patienten mit cT1-T3a-Prostatakarzinomen mit einem Gleason-Score von 8–10 bei jedem Serum-PSA-Wert, oder mit einem Gleason-Score von 7 bei einem PSA-Wert > 15 ng/ml, oder mit einem Gleason-Score von 6 bei einem PSA-Wert > 30 ng/ml. Weiter wurden cT3b-cT4-Karzinome ohne Gleason-Score- oder PSA-Wert-Begrenzung eingeschlossen. Auch wurden aktuelle Staging-Methoden eingesetzt. So bekamen 80 % der Patienten eine PSMA-PET/CT. Alternativ war ein konventionelles Staging mittels Abdomen- und Becken-CT sowie einer Knochenszintigraphie zulässig. In die Studie aufgenommene Patienten hatten im durchgeführten Staging keine Lymphknoten- oder Fernmetastasen. Die Effektivität einer elektiven Bestrahlung pelviner Lymphabflusswege nach der RTOG-Konsensus-Konturieranleitung zusätzlich zur dosiseskalierten Strahlentherapie der Prostata wurde in der POP-RT-Studie somit bei Patienten mit lokal fortgeschrittenem oder lokalisiertem Prostatakarzinom der Hochrisikogruppe untersucht. Das Risiko von Lymphknotenmetasen bei den eingeschlossenen Patienten betrug nach der Roach-Formel im Median 37,8 %, Interquartilenabstand 25–59 %. Die Hälfte der Patienten hatte ein Karzinom vom Gleason-Score 8–10. Zwei Drittel der Patienten hatten ein lokal fortgeschrittenes Karzinom der Kategorie T3a und T3b und 8 % ein T4-Karzinom. Die Strahlentherapie wurde mittels IMRT, VMAT oder Tomotherapie durchgeführt. Es wurde in einer Technik mit integriertem Boost bestrahlt. Auf die Prostata, jede extrakapsuläre makroskopische Tumorextension und die Samenblasenbasen wurde in einer moderaten Hypofraktionierung eine Gesamtdosis von 66–68 Gy in 25 Fraktionen in 5 Wochen appliziert. Bei einer Reparaturkapazität der Prostata, charakterisiert durch einen Alpha‑/Beta-Wert von 1,5 Gy, ist dies einer Gesamtdosis von 78–82 Gy am Tumor äquivalent, so dass die Strahlentherapie im aktuellen Dosisbereich durchgeführt wurde. Um die Prostata wurden PTV-Säume von 7 mm, jedoch nach dorsal von 5 mm eingehalten. Im Arm mit elektiver pelviner Lymphabflussbestrahlung („whole pelvic radiotherapy“, WPRT) wurde der pelvine Lymphabfluss außerhalb von Prostata und Samenblasen mit 2,0 Gy pro Fraktion bestrahlt. Die Gesamtdosis hier betrug dann 50 Gy. Alle Patienten erhielten eine neoadjuvante Hormonentzugstherapie (ADT) über mindestens 8 Wochen sowie eine adjuvante ADT, zusammen über insgesamt 24 Monate. Primärer Endpunkt der Studie war die BFFS (Freiheit vom biochemischen Rezidiv).

## Ergebnisse

In die POP-RT-Studie wurden 224 Patienten eingeschlossen. Die mediane Nachbeobachtungszeit betrug 68 Monate. Es wurde ein Vorteil für die WPRT auf dem primären Endpunkt, der Freiheit vom biochemischen Rezidiv, gefunden. Diese betrug nach der Kaplan-Meier-Methode mit WPRT 95,0 % nach 5 Jahren und ohne WPRT 81,2 % (*p* < 0,0001). Das Hazard-Ratio (HR) nach der Proportional-Hazard-Methode betrug 0,23 (95 % CI 0,10–0,50) zwischen den Armen mit WPRT und ohne WPRT. Mit WPRT wurden 7 biochemische Rezidive bei 110 Patienten, ohne WPRT 29 bei 112 Patienten gefunden. Der Endpunkt Gesamtüberleben (OS) wurde nicht verbessert (92,5 % vs. 90,8 % nach 5 Jahren; *p* = 0,83), wohl aber das fernmetastasenfreie Überleben nach 5 Jahren um 7 % nach WPRT (95,9 % vs. 89,2 % nach 5 Jahren; *p* = 0,01). Die kumulative Häufigkeit von Grad 2+ urogenitalen Toxizitäten war nach WPRT höher als nach alleiniger Strahlentherapie der Prostata (20 % vs. 8,9 %; *p* = 0,02).

## Kommentar

Trotz großer Unterschiede in der Freiheit vom biochemischen Rezidiv wurde bei moderater Nachbeobachtungszeit kein Vorteil bezüglich des Gesamtüberlebens gefunden. Dies deckt sich mit der Analyse der Intermediate Clinical Endpoints in Cancer of the Prostate Working Group. Diese fand, dass beim lokalisierten Prostatakarzinom mit intermediärem oder hohem Risiko sowie beim lokal fortgeschrittenen Prostatakarzinom das progressionsfreie Überleben nur ein schwacher Surrogatmarker für das Gesamtüberleben ist in Studien zur Strahlentherapie [4].

In der POP-RT-Studie wurde das PSA-Rezidiv als Ereignis für den primären Endpunkt verwendet. Dieses ist eng mit dem progressionsfreien Überleben (PFS) als frühem Endpunkt verknüpft, da in Strahlentherapiestudien zum Prostatakarzinom der Hochrisikogruppe mehr als 80 % der Ereignisse auf dem PFS-Endpunkt PSA-Rezidive sind [5]. Zunehmend besser werdende Salvage-Therapien beim oligorekurrenten Prostatakarzinom schwächen die Relation zwischen beiden Endpunkten. Auch machen interkurrente, nicht tumorbedingte Todesfälle einen Großteil der Todesursachen in diesen Risikogruppen aus. Die Relation der absoluten Risikoreduktion auf dem Endpunkt Freiheit vom biochemischen Rezidiv beim lokal fortgeschrittenen Prostatakarzinom auf der einen und dem Gesamtüberleben auf der anderen Seite wurde in dem parallel erschienenen Review der Autoren zur POP-RT-Studie in *Der Urologe *untersucht [6].

Insgesamt folgt aus den Studien zum alleinigen Hormonentzug versus Hormonentzug und Strahlentherapie der Prostata oder der Prostata mit Lymphabfluss, der PR.3-PR7-Studie, der SPCG/SUFUO-3-Studie und einer französischen Studie von Mottet, dass bei durchschnittlich 5,9 vermiedenen PSA-Rezidiven innerhalb von 5 Jahren ein Todesfall innerhalb von 10 Jahren vermieden wird. Ein ähnlicher Zusammenhang besteht beim Mammakarzinom nach brusterhaltender Operation. In der POP-RT-Studie kann nach dieser 5,9:1-Relation aus einer um 13,8 % verbesserten Freiheit vom biochemischen Rezidiv nach 5 Jahren ein um etwa 2,3 % verbessertes Gesamtüberleben nach 10 Jahren erwartet werden. Dieser Effekt ist kleiner als der in der RTOG-0924-Studie durch eine zusätzliche WPRT erwartete Effekt (Identifier: NCT01368588 at www.Clinicaltrials.gov). Diese RTOG-Studie ist die bisher größte randomisierte Studie zur Effektivität der WPRT zusätzlich zur Strahlentherapie der Prostata und der Samenblasen, kombiniert mit einer Kurzzeit-ADT bei Patienten mit lokalisiertem Prostatakarzinom und ungünstigem intermediärem oder hohem Risiko sowie von ausgewählten Untergruppen von Patienten mit lokal fortgeschrittenem Karzinom. Endpunkt war das Gesamtüberleben. Die Rekrutierungsphase wurde beim Stand von 2592 eingeschlossenen Patienten bereits abgeschlossen. Mit einer Auswertung der Ergebnisse wird Mitte des Jahres 2027 gerechnet. Man darf einen Anstieg des OS nach 10 Jahren von 6,5 % unter der Alternativhypothese erwarten.

Nachteile der in der POP-RT-Studie verwendeten Technik des integrierten Boosts können sich bei der Bildführung der Strahlentherapie ergeben. Bei engen PTV-Säumen um die Prostata und Online-Navigation mittels CB-CT auf die Prostata werden größere PTV-Säume um das größere Volumen des pelvinen Lymphabflusses notwendig, die von der Lagevariabilität der Prostata im Becken beim einzelnen Patienten abhängig sind. Alternative ist der sequenzielle Boost mit initial etwas größeren Sicherheitssäumen um die Prostata während der Phase mit WPRT und anschließender Präzisionsbestrahlung auf die Prostata mit engen Sicherheitssäumen um die Prostata bei täglicher Online-Navigation. Bisher sind zwei randomisierte Studien zur WPRT abschließend publiziert. Beide zeigten keinen eindeutigen Vorteil der WPRT. In der GETUC-01-Studie war das Rückfallrisiko der Patienten nur moderat. Insgesamt hatten 50 % der rekrutierten Patienten ein Lymphknotenbefallsrisiko von < 15 % nach der Roach-Formel [2]. Eine ADT wurde nur in der Hochrisikogruppe gegeben. Ein Vorteil im Arm mit WPRT zusätzlich zur Strahlentherapie von Prostata und Samenblasen wurde mit einem PFS von 66,0 % vs. 65,3 % im Kontrollarm nach 5 Jahren nicht gefunden. Die RTOG-9413-Studie prüfte in einem 2 × 2-faktoriellen Design sowohl den Zeitpunkt des Starts der 4 Monate dauernden ADT, neoadjuvant 2 Monate, vor Beginn der Strahlentherapie oder adjuvant nach Beendigung der Strahlentherapie, als auch die Effektivität der WPRT. Die Patienten hatten ein lokalisiertes oder lokal fortgeschrittenes Prostatakarzinom mit einem Lymphknotenbefallsrisiko > 15 % [1]. Die Gesamtdosis auf Prostata und Samenblasen betrug 70,2 Gy mit 1,8 Gy pro Fraktion. Es wurde ein signifikanter Interaktionseffekt zwischen der WPRT und dem Zeitpunkt des Beginns der ADT gefunden, jedoch kein eindeutiger Vorteil der WPRT. Primärer Endpunkt auch in dieser Studie war das PFS. Das PFS betrug nach neoadjuvanter ADT und mit WPRT 29,4 % sowie bei adjuvanter Hormonentzugstherapie ohne WPRT 30,2 % nach 10 Jahren. Insgesamt zeigt diese Studie kein eindeutiges Bild vom Stellenwert der WPRT unabhängig von der Sequenz der ADT. Aber es fand sich im Arm mit neoadjuvanter ADT und WPRT eine gering höhere kumulative Inzidenz von Grad-3- und vereinzelten Grad-4-Nebenwirkungen im gastrointestinalen Bereich, verglichen mit anderen Therapie-Armen mit allerdings veralteter Strahlentherapietechnik.

Die absoluten Ergebnisse der POP-RT-Studie mit WPRT sind sehr günstig. Ähnliche Raten werden aber auch in anderen aktuellen Studien ohne WPRT erreicht. PFS-Raten von 84 % wurden für Patienten der Hochrisikogruppe in der schwedischen Studie zur Ultrahypofraktionierung ohne Hormonentzugstherapie erzielt [5]. Zwei konsekutive prospektive Studien aus Toronto zur stark hypofraktionierten Strahlentherapie beim lokalisierten Hochrisiko-Prostatakarzinom zeigten kumulative Inzidenzen von biochemischen Rezidiven von unter 15 % im Verlauf von 5 Jahren nach Strahlentherapie der Prostata inklusive der basalen Samenblasenanteile mit und ohne WPRT [7]. In dieser Studie wurde ein Hormonentzug von 12–18 Monaten durchgeführt. Die niederländische FLAME-Studie mit einem Anteil von Hochrisikopatienten von 84 % zeigte weiterhin, dass sich die PSA-Rezidivfreiheit nach 5 Jahren bei Patienten mit Hochrisiko-Prostatakarzinom auch mit einem intraprostatischen Boost zusätzlich zur hochdosierten Strahlentherapie der Prostata und Samenblasen von 85 % auf 92 % signifikant steigern lässt (Hazard-Ratio = 0,45; *p* < 0,0001; [8]). Bei längerer Nachbeobachtungszeit über 5 Jahre hinaus ist bei Patienten der Hochrisikogruppe in allen Studien mit begrenzter Nachbeobachtungszeit mit einem weiteren Anstieg der Inzidenz von PSA-Rezidiven zu rechnen.

Eine vielversprechende alternative Strategie zur WPRT im Rahmen der Primärtherapie ist die ablative Lokaltherapie von oligorekurrenten Rezidiven lymphonodulär oder auch ossär, unterstützt von sensitiven Methoden der Rezidivdetektion wie der PSMA-PET/CT [9]. Mit einer stereotaktisch ablativen Salvage-Strahlentherapie oder Operation kann bei nodal-oligorekurrenter Erkrankung ein metastasenfreies Überleben von 50 % nach 5 Jahren erreicht werden [10]. Auch existieren im erneuten PSA-Rezidivfall zunehmend wirksame antihormonelle Strategien, die zu einem besseren Gesamtüberleben führen. Kombinationen aus modernen antihormonellen Therapien, PSMA-PET-Bildgebung und einer Salvage-Strahlentherapie im hormonsensitiven Rezidiv werden aktuell in weiteren Studien untersucht, beispielsweise PRIMORDIUJM [11].

## Fazit

Die Evidenz zur Effektivität der WPRT bei Patienten mit lokal fortgeschrittenem und lokalisiertem Prostatakarzinom der Hochrisikogruppe wird durch die POP-RT-Studie auf dem Endpunkt Freiheit vom PSA-Rezidiv nach 5 Jahren deutlich gestärkt. Bei Einsatz von modernen Staging-Untersuchungen, einer dosiseskalierten Strahlentherapie und durch begleitende ADT kann eine Freiheit von PSA-Rezidiven bei 95 % der Patienten erreicht werden. Ein Vorteil beim Gesamtüberleben scheint sich allerdings in der POP-RT-Studie nicht einzustellen. So kann eine generelle Empfehlung zur WPRT nicht ausgesprochen werden.

Beim individuellen Patienten mit einem sehr hohen PSA-Rezidivrisiko kann dieses Risiko durch die WPRT nach der POP-RT-Studie deutlich gesenkt werden, was insbesondere bei jungen Patienten mit geringen interkurrenten Begleiterkrankungen eine hohe Bedeutung hat. Somit sollte die WPRT im Einzelfall bei diesen Patienten der Hochrisikogruppe erwogen werden. Die Toxizität der WPRT scheint nur moderat, aber nachweisbar zu sein.

Für die interdisziplinäre Beratung hat die hier kommentierte Studie einen hervorgehobenen Stellenwert.

*Martin Stuschke und Boris Hadaschik, Essen*
